# Modeling the Violation of Reward Maximization and Invariance in Reinforcement Schedules

**DOI:** 10.1371/journal.pcbi.1000131

**Published:** 2008-08-08

**Authors:** Giancarlo La Camera, Barry J. Richmond

**Affiliations:** Laboratory of Neuropsychology, National Institute of Mental Health, National Institutes of Health, Department of Health and Human Services, Bethesda, Maryland, United States of America; University College London, United Kingdom

## Abstract

It is often assumed that animals and people adjust their behavior to maximize reward acquisition. In visually cued reinforcement schedules, monkeys make errors in trials that are not immediately rewarded, despite having to repeat error trials. Here we show that error rates are typically smaller in trials equally distant from reward but belonging to longer schedules (referred to as “schedule length effect”). This violates the principles of reward maximization and invariance and cannot be predicted by the standard methods of Reinforcement Learning, such as the method of temporal differences. We develop a heuristic model that accounts for all of the properties of the behavior in the reinforcement schedule task but whose predictions are not different from those of the standard temporal difference model in choice tasks. In the modification of temporal difference learning introduced here, the effect of schedule length emerges spontaneously from the sensitivity to the immediately preceding trial. We also introduce a policy for general Markov Decision Processes, where the decision made at each node is conditioned on the motivation to perform an instrumental action, and show that the application of our model to the reinforcement schedule task and the choice task are special cases of this general theoretical framework. Within this framework, Reinforcement Learning can approach contextual learning with the mixture of empirical findings and principled assumptions that seem to coexist in the best descriptions of animal behavior. As examples, we discuss two phenomena observed in humans that often derive from the violation of the principle of invariance: “framing,” wherein equivalent options are treated differently depending on the context in which they are presented, and the “sunk cost” effect, the greater tendency to continue an endeavor once an investment in money, effort, or time has been made. The schedule length effect might be a manifestation of these phenomena in monkeys.

## Author Summary

Theories of rational behavior are built on a number of principles, including the assumption that subjects adjust their behavior to maximize their long-term returns and that they should work equally hard to obtain a reward in situations where the effort to obtain reward is the same (called the invariance principle). Humans, however, are sensitive to the manner in which equivalent choices are presented, or “framed,” and often have a greater tendency to continue an endeavor once an investment in money, effort, or time has been made, a phenomenon known as “sunk cost” effect. In a similar manner, when monkeys must perform different numbers of trials to obtain a reward, they work harder as the number of trials already performed increases, even though both the work remaining and the forthcoming reward are the same in all situations. Methods from the theory of Reinforcement Learning, which usually provide learning strategies aimed at maximizing returns, cannot model this violation of invariance. Here we generalize a prominent method of Reinforcement Learning so as to explain the violation of invariance, without losing the ability to model behaviors explained by standard Reinforcement Learning models. This generalization extends our understanding of how animals and humans learn and behave.

## Introduction

In studying reward-seeking behavior it is often assumed that animals attempt to maximize long term returns. This postulate often forms the basis of normative models of decision making [1], choice behavior [2]–[4], and motivation [5], and plays a prominent role in the field of Reinforcement Learning (RL; see, e.g., [6]). RL is a set of methods for learning to predict rewarding outcomes from their association with environmental cues, and to exploit these predictions to generate effective behavioral policies. These are policies that comply with principles of reward maximization [7],[8] and invariance [9],[10]. Applied to reward-seeking behavior, the principle of reward maximization states that subjects should maximize the reward/cost ratio, and the invariance principle that subjects should be equally motivated when facing situations with identical reward/cost ratios.

The idea of maximizing reward over time or effort is general and has provided an effective basis for describing decision-making where the choice between available options is basically a matter of preference. RL methods such as the method of temporal differences (TD) constitute an efficient way of solving decision problems in tasks where a subject must choose between a larger vs. a smaller reward, or between a more probable vs. a less probable reward, and predict courses of actions comparable to the actual behavior observed in animals performing the same tasks [11]–[13].

**Figure 1:**
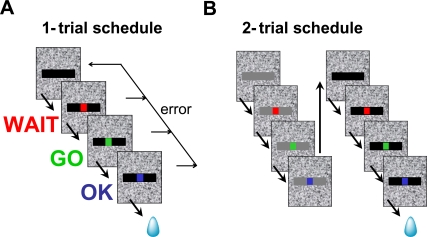
Behavioral paradigm used in the reward schedule task. (A) Color discrimination task. Each trial begins with the monkey touching a bar. A visual cue (horizontal black bar) appears immediately. Four hundred milliseconds later a red dot (WAIT signal) appears in the center of the cue. After a random interval of 500–1500 ms the dot turns green (GO signal). The monkey is required to release the touch-bar between 200 and 800 ms after the green dot appeared, in which case the dot turns blue (OK signal), and a drop of water is delivered 250 to 350 ms later. If the monkey fails to release the bar within the 200–800 ms interval after the GO signal, an error is registered, and no water is delivered. An anticipated bar release (<200 ms) is also counted as an error. (Red, green and blue dots are enlarged for the purpose of illustration). (B) 2-trial schedule. Each trial is a color discrimination task as in panel *A*, with cues of different brightness for different trials (see Materials and Methods for details). In the 2-trial schedule, completion of the first trial is not rewarded and is followed by the second trial after an inter-trial interval (ITI) of 1–2 seconds. An error at any point during a trial causes the trial to be aborted and then started again after the ITI interval. The same applies to schedules of any length. Schedules of different length are randomly interleaved. Note that after an error, the schedule is resumed from the current trial and not from the first trial of the schedule.

RL methods have proven less successful, however, in situations where motivation, defined as the incentive to be engaged in a task at all, plays a strong role [14]–[16]. A case in point is the behavior of monkeys performing visually-cued reinforcement schedules [17], wherein a series of identical actions is required to obtain reward, and a visual cue indicates how many trials remain to be completed before a reward is delivered (“reward schedule task,” see Figure 1). In this task, the error rate of the monkeys is proportional to the number of unrewarded trials remaining before reward, indicating that the value of the trial is modified by knowing the number of remaining trials. This violates the principle of reward-maximization: monkeys make errors in unrewarded trials that will have to be repeated, thus preventing optimal reward-harvesting behavior.

Here we show that in trials equally far from reward, monkeys make fewer errors in longer schedules, when more trials have already been performed (“schedule length effect”). Thus, the value of the current trial is also modified by the number of trials already completed. This behavior violates the principle of invariance: monkeys perform differently in trials equally far from reward, depending on the number of trials already completed in the current schedule. Taken together, these results suggest that the behavior in the reward schedule task does not develop under the principles of invariance and reward-optimization, as commonly assumed when applying RL methods to understanding reward-seeking behavior.

We present a RL rule which predicts the monkeys' behavior in the reward schedule task. Such a rule is a heuristic generalization of TD learning. When applied to the reward schedule task, it predicts all aspects of monkeys' behavior, including the sensitivity to the contextual effect due to schedule length leading to the violation of the invariance principle. When applied to a task involving choice preference, the new method predicts the same behavior as does the standard TD model. Thus, the behaviors in the reward schedule and in choice tasks can be the consequence of the same learning rule.

Building on the special cases of the reward schedule and choice tasks, we then provide a general theory for Markov Decision Processes, wherein the transition to the next state is governed in a manner similar to a choice task, but is conditioned on whether the agent is sufficiently motivated to act at all, like in the reward schedule task. Finally, we link the schedule length effect to instances of “framing” [18],[19] and “sunk cost” effects [20],[21], which also emerge in conjunction with the violation of the principle of invariance.

## Results

### Behavior

In this work we collate the behavior of 24 monkeys tested in the reward schedule task [17], and analyze the entire set of data as a group (see Material and Methods). In this task, a series of trials had to be completed successfully to obtain reward at the end of the series. This series is defined to be a schedule, which is then characterized by its length measured in number of trials (Materials and Methods and Figure 1). The monkey starts each trial by holding a bar which causes a visual cue to appear on a computer screen, followed by the appearance of a red dot in the middle of the screen. The monkey must wait for the red dot to turn green (“GO” signal), at which point it must release the bar within a 200–1000 ms window. If the bar is released correctly, the monkey proceeds to the next trial of the schedule. Each trial must be repeated until performed correctly.

**Figure 2:**
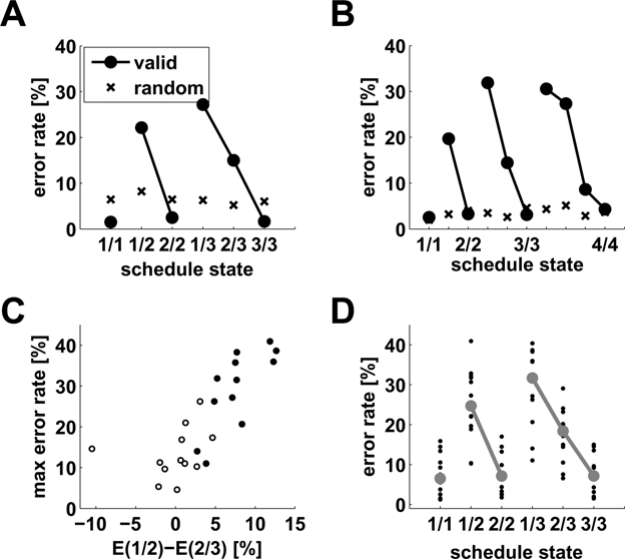
Monkeys' behavior in the reward schedule task. (A–B) Error rates as a function of schedule state for two monkeys, for both valid (circles) and random cues (“x”). Each schedule state is labeled by the fraction *τ*/*s*, where *τ* stands for current trial and *s* stands for current schedule length. Maximal schedule length was 3 for monkey A and 4 for monkey B. In both monkeys, error rates with valid cues are significantly different (*?* ^2^ test, *p*<10^−10^). In monkey A, the error rate in states 1/2 is larger than in state 2/3 (Marasquilo test for multiple comparisons, *p*<0.005); in monkey B, the error rate in state 2/3 is larger than in state 3/4, and error rate in state 1/2 is larger than in state 2/3 (Marasquilo test, *p*<0.05). Original data from refs. [25] (*A*) and [44] (*B*). (C) scatter plot of the difference in error rates between states 1/2 and 2/3 (*E*(1/2)−*E*(2/3)) vs. the maximal error rate for all 24 monkeys. Filled circles mark positive differences *E*(1/2)−*E*(2/3) which are significant (Marasquilo test for multiple comparisons, *p*<0.05). (D) error rates (dots) for the 12 monkeys corresponding to the closed circles in panel *C*. Thick grey lines: medians.

In the presence of visual cues informing the monkey of the progress through the schedule (Valid Cue condition), the percentage of errors in all monkeys was directly related to the number of trials remaining to be completed in the schedule, i.e., the largest number of errors occurred in the trials that are furthest from the reward (*?* ^2^ test, *p*<0.05; Figure 2A and 2B, circles; each trial is labeled by the fraction *τ*/*s*, where *τ* stands for current trial and *s* stands for current schedule length). The performance in terminal trials was indistinguishable across schedules for each monkey, was above 94% correct in 14 out of 24 monkeys, and above 90% in 19 out of 24 monkeys.

In the Random Cue condition the visual cues were selected at random and bore no relationship to schedule state. In such a condition, error rates were indistinguishable across all schedule states (or idiosyncratic; “x” in Figure 2A and 2B; *?* ^2^ test, *p*>0.05 in 10 out of 15 monkeys tested in the Random Cue condition), and close to the error rates in terminal trials in the Valid Cue condition. Thus, performance in unrewarded trials in the Valid Cue condition was well below the ability of the monkeys. Since the individual trials of each schedule have the same perceptual and motor demands, we interpret the different error rates as being related to the different levels of motivation. This interpretation is also supported by the observation that, in most monkeys, the reaction times become faster as the end of the schedule is approached [17], [22]–[25].

In the penultimate trials of each schedule (i.e., 1/2, 2/3, and 3/4 when available) 20 of 24 monkeys made progressively fewer errors as the schedule became longer (sign test, *p*<0.005). The error rate in state 1/2 was significantly larger than in state 2/3 in 12 out of 20 monkeys (Marascuilo procedure, *p*<0.05, see Materials and Methods and Figure 2C and 2D). In two of three monkeys tested with 4 schedules, the error rate in state 2/3 was also significantly larger than in state 3/4. The third monkey tested with 4 schedules showed small error rates, and multiple comparisons between penultimate trials were not significant (monkeys often will not perform the task with 4 schedules [17]).

In many of these studies the cues were distinguished by their brightness, where their brightness had been set according to the number of trials remaining in the schedule (Material and Methods), raising the possibility that performance was related to judging the brightness. However, this seems unlikely because the behavioral sensitivity was also seen when unique stimuli, e.g., Walsh patterns, were used as cues (e.g., Figure 2 of [26]), where no feature of the visual stimulus is a graded function of reward proximity or progress through the schedule. In conclusion, in a population of monkeys there was a significant tendency for motivation to increase with the number of trials already performed, at parity of proximity to reward. We refer this phenomenon to as the “schedule length effect.”

### Model

In the reward schedule task, all trials have the same cost because they all require the same action in response to the same trigger (the appearance of the green dot); trials differ only in their proximity to reward, which in turn does not depend on how many trials have already been performed. A standard reinforcement learning method can only learn to predict the proximity to reward correctly, and thus, unlike the behavior shown by the monkeys, is insensitive to the context introduced by the schedule length. We address this issue in detail in the remainder of this manuscript.

#### The basic model

We assume that performance, here measured as the percentage of correct trials in each schedule state, reflects the monkey's motivation, which in turn reflects the value of that schedule state. Both rewarded and unrewarded trials acquire value: if unrewarded trials had no value, the monkeys would not perform them because there would be no motivation to do so. Thus, value acquisition must be based on a mechanism capable of learning to predict delayed rewards, like the method of temporal differences [27]. The values of the trials reflect any attribute that will affect motivation such as temporal discounting or intrinsic reward value, and thus will be referred to as motivational values. Performance accuracy will be guided completely by the motivational values, whereas other factors such as sensory or motor thresholds will be ignored, given that all monkeys found the color-discrimination task required in each trial (Figure 1A) easy to learn and perform (see Materials and Methods).

The model below establishes the functional connection between performance and motivational values, and provides a recipe for learning the values. In general terms, the model assumes that the agent, on any given state *S*, performs the required action correctly with a probability proportional to the value of that state, *V*(*S*), through a softmax “performance function”:
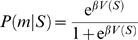
(1)


The parameter *β* controls the steepness of the performance function in the region around *V*(*S*) = 0. Thus, *P*(*m*|*S*) is the probability of being motivated enough to perform the action required to leave state *S*. Its complement, *P*(*m̅*|*S*), is the probability that the agent is not motivated enough to perform correctly, i.e., the model correlate of the error rate in state *S*:
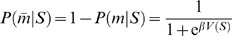
(2)


**Figure 3:**
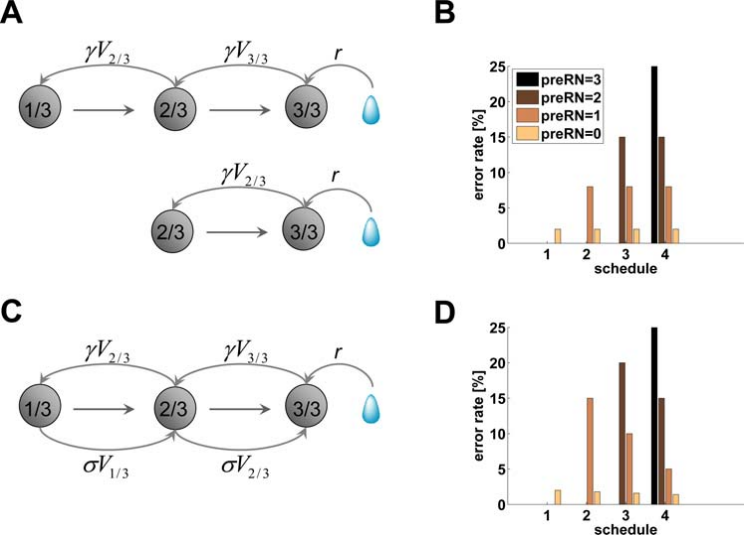
Models. (A) Diagrammatic representation of the basic model for 3- and 2-trial schedules. (B) General pattern of error rates predicted by the basic model. For trials with the same reward proximity (pre-reward number, *preRN*, plotted in the same color) the model predicts equal error rates. (C) Diagrammatic representation of the context-sensitive model for the 3-trial schedule. (D) General pattern of error rates predicted by the context-sensitive model. For trials with the same reward proximity (in same color) the model predicts smaller error rates in longer schedules.

In the particular case of the reward schedule, Equation 1 specifies completely the “policy” followed by the agent. We shall clarify in a later section that Equation 1 is a special case of the policy we propose for general Markov Decision Processes. *V*(*S*) is updated trial-by-trial according to
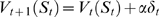
(3)where *t* is the trial number, *V_t_*_+1_(*S_t_*) is the value of current state *S_t_* at trial *t*+1, *α* is a learning rate (here a constant), and *δ* is the temporal difference term of TD learning:
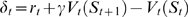
(4)
*V_t_*(*S_t_*_+1_) is the *current* value of the state expected to follow the action taken. *r_t_* is the reward delivered as a consequence of that action (*r* = 0 in all incorrect trials and in correct, unrewarded trials, and *r* = 1 in correct, rewarded trials). Parameter γ, with 0≤*γ*<1, is a temporal discount factor that establishes the importance of the next state's value for updating the value of the current state (Figure 3A). When *γ* = 0, the value of the current state is only related to the immediate reward contingency; when *γ*>0, *all* future contingencies affect the value of the current state, weighted by proximity, allowing learning to predict delayed contingencies, see, e.g., [6]. Learning is accomplished by minimizing the difference *δ_t_*. When an error is made, a negative *δ* follows, decreasing the value of the current state, thereby increasing the probability of making an error upon the next occurrence of that state. By performing correctly (by chance or otherwise), the algorithm reinforces the values of the schedule states, increasing the probability of correct performance in the future. At equilibrium, the average *δs* vanish in each schedule state, and the state-values fluctuate around their equilibrium values. So far the main difference from the more common implementations of TD learning is that these latter implementations use *δ* also to improve the action selection process directly, whereas here the parameters of the performance function (Equation 1) are held constant. When the TD signal *δ* is also used to improve the action selection process, this typically leads to the development of an optimal policy, i.e., a policy which maximizes the long-term acquisition of reward [6]. Following Sutton [27], we interpret TD learning as a general means of learning to predict delayed contingencies, and not necessarily for learning an optimal policy.

In the reward schedule, a state is defined by the pair {*τ*,*s*}, where *τ* stands for current trial and *s* stands for current schedule length, so that Equation 4 reads
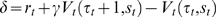
(5)If an error is made, *τ*+1 is replaced by *τ* because the trial is repeated. In a correct terminal trial (*τ* = *s* and *r_t_*>0), the next state *S_t_*_+1_ is unknown, and its value is set to zero (the common choice in RL, see, e.g., [6]). An alternative option would be to endow the model with some “post-reward expectation” and assign some positive value to the next state (for example, the mean value of the first trials). This approach scales all values by a constant factor, producing the same qualitative behavior (see Materials and Methods for details).

In the validly-cued reward schedule, learning continues until the average *δ* vanishes in all states. In terminal trials, *V* = *r*, where *r* is the amount of reward received in each rewarded trial. For non-terminal trials (*τ*<*s*), the equilibrium condition is *V*(*τ*,*s*) = *γV*(*τ*+1,*s*), i.e., trials more proximal to reward are more valuable, and by iteration
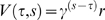
(6)Here, *V* depends only on the difference between *s* and *τ*, i.e., the proximity to reward. Therefore, it can be written as
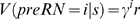
(7)where *preRN* = *s*−*τ* is the pre-reward number (defined to be zero in terminal trials), and *V*(*i*|*s*) is the value of the state having *preRN* = *i*, conditioned on schedule length being *s* (which, as Equation 7 shows, does *not* depend on *s*). Trials with larger *preRN* have smaller values (since *γ*<1). The actual values will converge to the theoretical values given above only in the absence of errors, i.e., if the policy is to execute every trial correctly independently of its value. If the policy is given by Equation 1, then the correct values must be found by solving self-consistent equations (given in the Materials and Methods, Equation 13). However, the results are qualitatively the same and for simplicity we shall use the values of Equation 7 in the following.

As Equation 7 shows, trials equally distant from reward will acquire the same value and thus produce the same error rate under any policy (Figure 3B). Thus, the basic model is not sensitive to the contextual effect of schedule length observed in the data, i.e., the difference in performance between penultimate trials belonging to schedules of different length. This phenomenon can be seen as a broken symmetry between trials with the same proximity to reward, which are equivalent in the basic model. In the next section we propose a different TD learning rule which is sensitive to the context produced by schedule length.

#### Context-sensitive model: the effect of schedule length

In the schedule length effect, the value of each trial is larger than predicted by proximity to reward alone. A simple speculation on how this might arise is that the value of each trial is enhanced by having completed any previous trial in the current schedule. This idea can be implemented by modifying the temporal difference rule as follows:
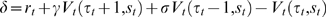
(8)In this rule, the value of the immediately preceding trial is also taken into account. The parameter *σ* quantifies the fraction of value carried forward to the next trial, with 0<*σ*<1. This is just the basic model when *σ* = 0. In first trials we set *σ* = 0, i.e., the value of rewarded trials is not carried forward. This seems a natural choice because the terminal states of each schedule are not part of the next sequence required to obtain reward. However, no qualitatively different behavior would result from keeping *σ*>0 in first trials also (see Materials and Methods). All other details are as in the basic model, including Equation 3 which remains unchanged. In this rule, a trial acquires value due to both the next trial and the previous one (Figure 3C). When learning has occurred (i.e., *δ* fluctuating around zero), the new learning rule gives *V*(*τ*,*s*) = *r*+*γV*(*τ*+1,*s*)+*σV*(*τ*−1,*s*) (compare with the value in the basic model, *r*+*γV*(*τ*+1,*s*)). This gives *V*(*τ*,*s*) a dependence on schedule length *s*, unlike Equation 7. In the absence of errors, the equilibrium values for the first three schedules are:
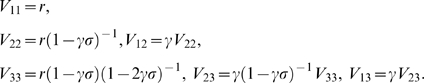
(9)If errors are made according to Equation 2, a more involved set of self-consistent equations defines the values implicitly (see Materials and Methods), but this does not affect the qualitative pattern of behavior described next. The relation *V*_1*s*_ = *γV*_2*s*_, that in the basic model holds for the values of all pairs of successive schedule states, applies here only to first trials, whereas the value of the intermediate trial *V*_23_ is augmented by a factor (1−*γσ*)^−1^>1 due to the temporal accumulation of values of past trials. This gives *V*_23_>*V*_12_, i.e., the value of state 2/3 is greater than that of state 1/2, as observed in the experiments. In general, the error rates will be different in trials with the same *preRN* but belonging to different schedules (Figure 3D). We refer to this model as “context-sensitive” because the motivational context due to the schedule length is an emergent property of, and not an input to, the model itself (e.g., through a redefinition of the schedule states). For *σ* = 0, Equation 6 of the basic model reappears.

#### Predictions of the models in the Random Cue condition

The model predicts equal values for random cues that bear no relationship to schedule state. This results in uniform error rates, with a small spread around the mean due to the stochasticity in the cue selection and the learning processes. Any TD-based model would make the same prediction, which is a consequence of a symmetry contained in the design of the task, i.e., all cues are associated with reward with the same frequency. In the absence of errors, the mean value of random cues is
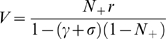
(10)where 0<*N*_+_<1 is the fraction of rewarded trials, and is equal to the number of schedules divided by the number of schedule states (Materials and Methods). Uniform performance is indeed observed in the data (black crosses in Figure 2A and 2B). Thus, the context-sensitive model can explain all the qualitative properties of the behavior in the reward schedule task.

**Figure 4:**
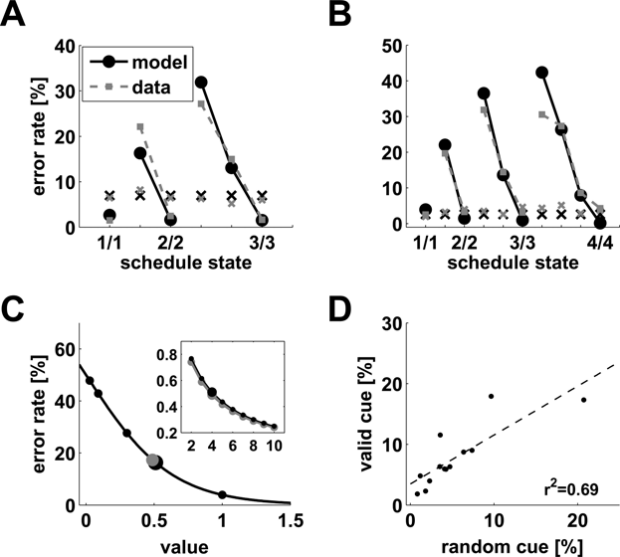
Predictions of the context-sensitive model in the reward schedule task. (A–B) Theoretical error rates predicted by the context-sensitive model (black) for both valid (circles) and random (“x”) cues. The model parameters were tuned to match the experimental error rates of Figure 2A and 2B respectively using least-square minimization as described in Materials and Methods. The experimental data from Figure 2 are reproduced in grey for comparison. Parameters for Monkey A (B): *β* = 3.6 (3.2), *σ* = 0.3 (0.8), *γ* = 0.4 (0.3). (C) Error rate (Equation 2) as a function of schedule state values (full curve) for the model of panel *B*. Black dots are the actual values of valid cues in the standard model (i.e., with *σ* = 0; see Equation 6); larger dots are the mean values of valid (black) and random (grey) cues. The inset shows the predicted mean values of valid (black) and random (grey) cues for paradigms with 2 to 10 schedules (basic model). Larger dots correspond to the case of main figure (4 schedules). (D) Linear regression of the median error rates with valid cues against the median error rates with random cues for the 13 monkeys tested in both conditions (*r* ^2^ = 0.69, *p*<0.0005).

The same properties can also be captured quantitatively, as shown by the best fits of the context-sensitive model to the data (Figure 4A and 4B). The parameters of the model were tuned to minimize the difference between the model fits and the experimental error rates (see Materials and Methods), and the same parameters values were used for valid and random cues. The best-fit values of *γ* and *σ* ranged from 0.2 to 0.8 and from 0.1 to 0.8, respectively (24 monkeys), and were strongly anti-correlated (*r* = −0.82, *r* ^2^ = 0.67, *p*<10^−6^). The negative correlation between *σ* and *γ* is to be expected since *σ* is a measure of the strength of the schedule length effect, which is stronger when the maximal error rate is larger (Figure 2C), and the latter is in turn inversely related to the discount rate *γ*. We emphasize that although we fit the model to individual experimental cases, the qualitative behavior predicted by the model is independent of the actual choice of parameters (provided that the value of *σ* is within the allowed range, see Materials and Methods).

#### Predictions of the context-sensitive model in the reward schedule task

Both the basic and context-sensitive models predict that valid and random cues have roughly the same average value, as shown in Figure 4C, which depicts the error rate as a function of the values of schedule states (Equation 2). The larger dots represent the mean values of valid (black) and random (grey) cues respectively, which are generally close to each other. In the inset of Figure 4C this is shown for 2 to 10 schedules. The mean values decrease with the number of schedules, since the average fraction of rewarded trials also decreases with the number of schedules. The prediction that valid and random cues have approximately the same average value does not have a direct experimental correlate, since one only has access to the error rates. Intuitively, however, the correlation between the mean values of valid and random cues should be reflected in the correlation between the median error rates in the two paradigms. This correlation was present in the data (Figure 4D, *r* ^2^ = 0.69, *p*<0.0005).

Figure 4C suggests that the overall level of motivation (as measured by the average values) is approximately the same with either valid or random cues. The non-linearity of the performance function explains why the overall performance is better in the Random Cue condition than in the Valid Cue condition. The values of valid cues (black dots in Figure 4C) spread around their mean (larger black dot), producing distributed error rates which depend on schedule state, whereas random cues' values have a limited spread (not shown) around their mean (grey dot) due only to random fluctuations, producing indistinguishable error rates. As shown in Figure 4C, the complement of the performance function (Equation 2) tends to flatten towards larger values more than for smaller ones. As a consequence, performance with random cues is much closer to the performance in validly cued rewarded, rather than unrewarded, trials (as, e.g., in Figure 2A and 2B).

#### Predictions of the context-sensitive model in choice tasks

To be a valid generalization of TD learning, the context-sensitive model must predict the same qualitative behavior is situations where animal behavior is well described by the standard model. We show here that this is generally true in situations involving behavioral choices. In particular, this means that the context-sensitive model does not predict a suboptimal behavior in tasks where this is not observed.

**Figure 5:**
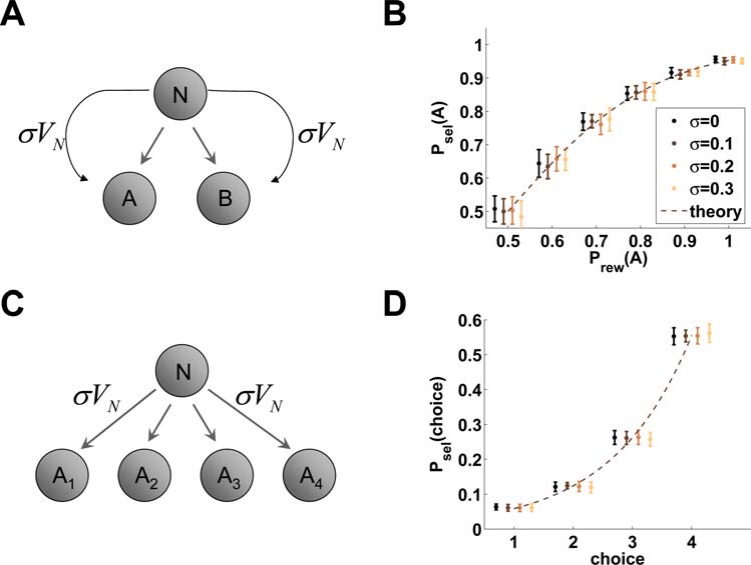
Predictions of the context-sensitive model in choice tasks. (A) Two-choice task. At decision node *N* (of value *V_N_*) the agent can either choose action *A* (which gives a larger or more probable reward) or action *B* (smaller or less probable reward). The same value *σV_N_* is carried over to whatever outcome of the choice (curved arrows). (B) Mean frequency of choosing action *A* in the two-choice task of panel *5A* (*P*_sel(*A*)_) vs. the probability that action *A* is rewarded (*P*_rew(*A*)_) for different values of *σ* (see the text). For each value of *P*_rew_(*A*), four values of *σ* were used (0. 0.1, 0.2, and 0.3). Shown are means (dots) and standard deviations (error bars) over 20 simulations with *β* = 3 and *r* = 1 together with the theoretical prediction 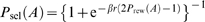 (dashed line). For *σ* = 0, the model is the standard TD model. Choice preference does not depend on the value of *σ*. (C) 4-armed bandit task. At decision node *N* the agent can choose between 4 possible actions, each rewarding the agent according to a predefined probability distribution. The same value *σV_N_* is carried over to whatever outcome of the choice. (D) Mean frequency of choosing each of the four alternative actions of the 4-armed bandit task of panel *5C* for different values of *σ* (same values as in panel *5B*). Each choice was rewarded according to a Gaussian distribution truncated at negative values, with mean *μ* = 0.25, 0.5, 0.75, 1 and standard deviation 0.25. Shown are means (dots) and standard deviations (error bars) over 20 simulations with *β* = 3, together with the theoretical prediction 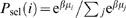 (dashed line). Choice frequencies do not depend on the value of *σ*.

A simple choice task entails the offer of two alternative options, say *A* and *B*, to an agent which has the freedom to choose between the two and will get rewarded accordingly. In the deterministic version of this task, one option is always rewarded, the other is never rewarded; in the probabilistic version, one of the options is rewarded more often than the other, and neither of them is rewarded with certainty; or both options are rewarded with equal probability but in different amounts; and so on (Figure 5A). A standard reinforcement model would predict a preference (as measured by choice ratio) for the more rewarded option in all cases, and will learn to choose always the rewarded option in the deterministic choice task. The context-sensitive model predicts exactly the same behavior (Figure 5B). This is most easily explained in the fully deterministic case where option *A* gives always a reward *r* whereas option *B* gives no reward (i.e., *P*_rew(*A*)_ = 1 in Figure 5B). In this case, for *σ* = 0 the values of the two options approximate the amount of reward obtained, i.e., *V_A_* = *r* and *V_B_* = 0. When 0<*σ*<1, the value of each option is increased on average by the same amount, i.e., 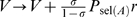, where *P*_sel(*A*)_ is the observed frequency of selecting *A*. Under the standard assumption that the action with larger value is selected more often, such preference will not be reversed in the context-sensitive model. For example, under a softmax model for *P*_sel(*A*)_, e.g., 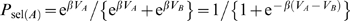, the probability of selecting *A* depends only on the difference *V_A_*−*V_B_*, which is left unchanged. Similar results are found for the probabilistic version of the task, where the best option is rewarded with probability *P*_rew(*A*)_>0.5 and the other with probability 1−*P*_rew(*A*)_ (Figure 5B). The two models give the same results also under a “greedy” policy, which by definition selects always the action with larger value (this can be obtained from the softmax function by letting *β*→∞). These conclusions hold also for variations of this two-choice task where the probability that one choice is the better option increases with the number of consecutive selections of the alternative choice, typically resulting in matching behavior (see, e.g., [28]; not shown).

The argument can be generalized to an *n*-choice task (Figure 5C), where each choice gives a reward drawn from a given distribution (sometimes called the *n*-armed bandit task in the RL literature, see, e.g., [6]). Also in this case, all values are increased by the same amount *c* in the context-sensitive model, and the softmax function,
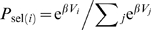
(11)is invariant under the scaling *V_j_*→*V_j_*+*c*. As a consequence, the frequency of each choice does not depend on the value of *σ* (Figure 5D).

**Figure 6:**
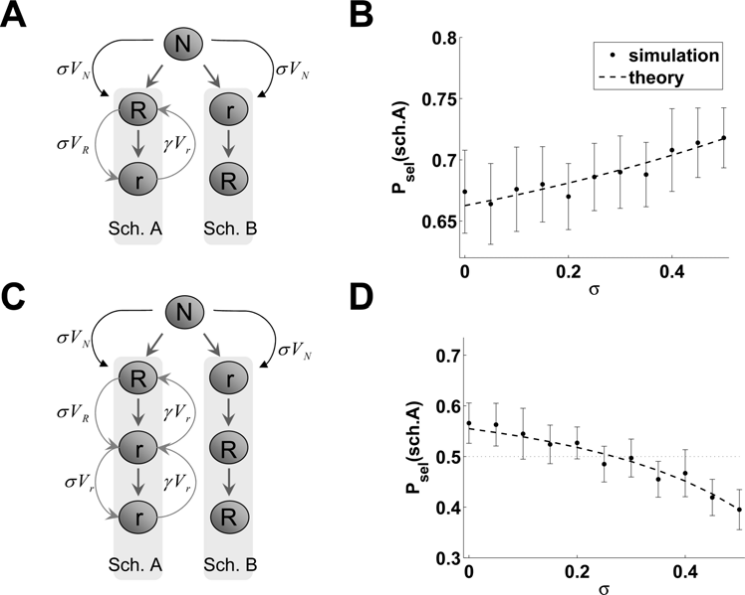
Predictions of the context-sensitive model in choice-schedule tasks. (A) Description of the choice-schedule task with 2-trial schedules. At decision node *N* (of value *V_N_*) the agent can either choose the immediate-reward schedule *A* (which gives a larger reward, *R*, sooner and a smaller reward, *r*, later) or the delayed-reward schedule *B* (smaller reward sooner and larger reward later). The same value *σV_N_* is carried over to whatever outcome of the choice, but following trials in each schedule modify the value of *A* or *B* differently (curved grey arrows, shown for schedule *A* only. See the text for details). (B) Mean frequency of choosing the immediate-reward schedule (schedule *A*) in the task of panel *6A* predicted by the model as a function of *σ*. Shown are means (dots) and standard deviations (error bars) over 20 simulations with *β* = 3, *γ* = 0.55, *R* = 1 and *r* = 0.5. Dashed line: theoretical prediction according to the equation 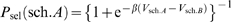 with *V*_sch.*A*_−*V*_sch.*B*_ = (1−*γσ*)^−1^(1−γ)(*R*−*r*). A positive value of *σ* enhances the existing preference for the immediate-reward schedule. (C) Choice-schedule task between two 3-trial schedules, a generalization of the task in panel *6A*. (D) mean frequency of choosing the immediate-reward schedule (schedule *A*) in the task of panel *6C* predicted by the model as a function of *σ*. Shown are means (dots) and standard deviations (error bars) over 20 simulations with the same parameters as in *6B*. Dashed line: theoretical prediction according to the equation 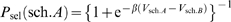 with *V*_sch.*A*_−*V*_sch.*B*_ = (1−2*γσ*)^−1^(1−*γ*−*γ* ^2^−*γσ*)(*R*−*r*). Dotted line: indifference point *P*_sel_(sch.*A*) = 0.5, i.e., the situation where the agent has no preference for either schedule. For *σ* larger than ≈0.268, choice preference is reversed and the delayed-reward schedule is chosen more often.

The reason for which a positive *σ* will not make a difference in a choice task is that at each decision node the same value (*σV_N_* in Figure 5A and 5C) will be carried over to whatever the outcome of the choice. For *σ*>0 to have an effect on the choice, the choice must be followed by a sequence of states or actions, with different amounts of previous value carried over in different sequences. To illustrate this point, consider a combination of a choice task and a reward schedule task which we shall name “two-choice schedule task” (Figure 6A). Assume that an agent can choose whether to receive a reward *R* now and a smaller reward *r* later on ({*R*,*r*}, schedule *A*), or the smaller reward now and the larger one later on ({*r*,*R*}, schedule *B*). This task is more complex than the simple choice task, because here the initial action or choice affects the return obtainable from subsequent ones. A standard TD model predicts a preference for schedule *A*, since this model discounts later rewards, penalizing reward *R* in schedule *B* more than in schedule *A*. Numerically, *V*_sch.*A*_−*V*_sch.*B*_ = (1−*γ*)(*R*−*r*)>0, and the “immediate-reward” schedule *A* is chosen more often than the “delayed-reward” schedule *B*. This is modified into *V_σ_*_;sch.*A*_−*V_σ_*_;sch.*B*_ = (1−*γσ*)^−1^(1−*γ*)(*R*−*r*)>*V*_sch.*A*_−*V*_sch.*B*_ in the context-dependent model. The difference between the values of the two schedules not only keeps its sign, but is increased in magnitude. This is because in the immediate-reward schedule *A*, a larger value is carried over to the next trial compared to the delayed-reward schedule. Thus, the context-sensitive model amplifies the existing preference for the immediate-reward schedule (Figure 6B). However, no new qualitative behavior emerges.

These conclusions hold for any choice schedule with two schedule states (i.e., with any choice of rewards and parameters values). In this more general case, parameters can be chosen so that a preference for either schedule could emerge; however, a positive value of *σ* can only amplify the existing preference (not shown).

A positive *σ* can alter an existing preference if the schedules comprise more than two trials. Consider for example the case where upon selection of schedule *A* the agent receives reward *R* at the first step and then a smaller reward *r* at the next two steps, i.e., a schedule of type {*R*,*r*,*r*}; whereas schedule *B* gives the smaller reward first, followed by the larger reward in the next two steps: {*r*,*R*,*R*} (Figure 6C). Since in this case *V*_sch.*A*_−*V*_sch.*B*_ = (1−*γ*−*γ* ^2^)(*R*−*r*), the immediate-reward schedule (schedule *A*) will be preferred only if 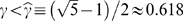. Depending on the value of the discount factor *γ*, both schedules may be preferred in this task under standard TD learning. For *γ* just above its critical value *γ*ˆ, a positive value of *σ* can only increase the agent's preference for the delayed-reward schedule *B*; but for *γ* just below its critical value *γ*ˆ, a positive value of *σ* can bias preference away from the immediate-reward schedule *A* and towards the delayed-reward schedule *B*. This is shown in Figure 6D, where *γ* = 0.55, *R* = 1, and *r* = 0.5. With these parameters, a reversal in preference occurs at *σ*≈0.268. It can be shown that for any choice schedule task with three states, a preference for the delayed-reward schedule will always be amplified by a positive *σ*, whereas a preference for the immediate-reward schedule could be reversed if some conditions are met. However, either schedule could be favored in the standard model also, depending on the value of the discount factor *γ*. In a choice task between two *n*-trial schedules, of type {*R*,*r*,*r*,…,*r*} and {*r*,*R*,*R*,…,*R*}, respectively, the critical value of *γ* is given by the real solution of 1−*γ*−*γ* ^2^−…−*γ^n^* ^−1^ = 0. This value decreases with *n* and approaches 0.5 when *n* tends to infinite (practically, for *n*>10). Since preference for one schedule or the other can be obtained in the standard model by adjusting the value of *γ*, there is no new qualitative behavior emerging from the context-sensitive model in this task. We conclude that in simple choice tasks and in choice-schedule tasks the context-sensitive model predicts the same qualitative behavior as the standard TD model.

#### General model for Markov Decision Processes (MDPs)

**Figure 7:**
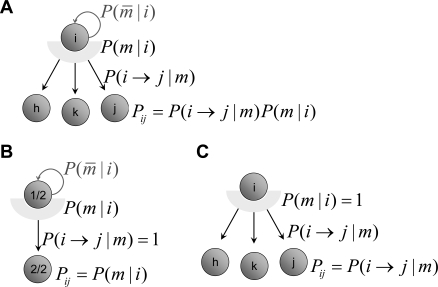
Model for the general Markov Decision Process (MDP). (A) Policy for the general MDP. In the fragment of MDP shown, the agent is in state *i* and must decide (1) whether to leave the state (with probability *P*(*m*|*i*)), and (2) in which state to go in case of a positive decision (weighting each choice with probability P(*i*→*j*|*m*)). Decision 1 depends on the motivational value of current state; decision 2 depends on the relative values of the possible arrival states, or choices. Both the motivational and the choice values are learned with the TD method of the main text. If the agent is not motivated to perform the trial, it will find itself in the same state one time step later (curved arrow). If the agent is sufficiently motivated to perform the trial correctly, it proceeds to make a choice. In the figure, this situation is represented by the curved shaded region from which the arrows to the possible choices reach out. In the general case, the transition probability *P_ij_* is the product of the probabilities *P*(*m*|*i*) and *P*(*i*→*j*|*m*). (B) Policy in the reward schedule task. In this case, *P*(*i*→*j*|*m*) because there is no choice and *j* can only be the next schedule state (in this example, *i* = 1/2, *j* = 2/2). Thus, *P_ij_* = *P*(*m*|*i*). (C) Policy in the choice task when considering only correct trials. In this case, *P*(*m*|*i*) is determined to be 1 and thus *P_ij_* = (*i*→*j*|*m*).

Reinforcement schedules and choice tasks are examples of MDPs. Formally, a MDP is a collection of states, each with an associated cost or reward, and a set of transition probabilities that govern the transitions between those states. We shall indicate with *P_ij_* the probability to move to state *j* from state *i* (Figure 7A). The numbers *P_ij_* must satisfy a number of properties, among which: (a) the transition from *i* to *j* must depend on current state *i* but not on any state previously visited (the Markov property), and (b) starting from *i*, a transition to some other state must occur, i.e., ∑*_j_ P_ij_* = 1 (see, e.g., [6] for an introductory exposition of the theory of MDPs). A MDP is a very general framework and is widely used as an abstract setting for RL problems [6], where the transition probability rule *P_ij_* is called a “policy.” However, this framework does not consider the effort due to an instrumental action required to leave the current state, which could induce some motivational reluctance to the subject, as shown in the reward schedule. This is also true in choice tasks, where even in the case of excellent performance, the percentage of correct trials is typically less than 100%. In defining a policy for the reward schedule (Equation 1) we have, in effect, introduced an example of what we shall call “instrumental MDP,” i.e., a MDP where each transition is conditioned on an instrumental action being performed correctly, otherwise an error results and no transition is possible, represented by a self-link to the current state in Figure 7A. We show in this section that a general policy can be introduced for an instrumental MDP, of which the policy Equation 1 for the reward schedule task, and the softmax function Equation 11 used in the choice tasks of Figures 5 and 6 are special cases.

In the more general case of an instrumental MDP, we shall define the policy, i.e. the probability of making a transition from *i* to *j*, as the product of the probability *P*(*m*|*i*) of leaving the current state *i* (by performing correctly), times the probability *P*(*i*→*j*|*m*) of transitioning to state *j*, given that the agent is motivated to do so:
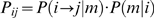
(12) (Figure 7A). In the reward schedule, *P*(*i*→*j*|*m*) equals 1 if *j* is the next state in the schedule, otherwise it equals zero, hence the policy reduces to *P_ij_* = *P*(*m*|*i*) (Figure 7B). In a choice task, the full policy Equation 12 should be used, which takes in account also the fraction of incorrectly performed trials. However, choice behavior is typically analyzed on correct trials only (as we have done in the previous section), since these are the trials where an actual choice occurs. On the subset of correct trials, *P*(*m*|*i*) is determined to be 1, and the policy reduces to *P_ij_*(*i*→*j*|*m*) (Figure 7C). This explains our use of different policies for reinforcement schedules and choice tasks, respectively.

Regarding the specific choice of policies we have adopted, note that *P*(*i*→*j*|*m*) need not be a softmax function 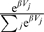 as in Figures 5 and 6, but could be any other suitable policy (e.g., a greedy or an ε-greedy policy, see [6] for details). As for *P*(*m*|*i*), the reward schedule data presented in this manuscript imply that *P*(*m*|*i*) cannot be taken to be the choice type, i.e., of the same type as *P*(*i*→*j*|*m*), regardless of the actual functional form used for it. This point can be illustrated with the following simple argument. Since error trials must be repeated, one might be tempted to frame the motivational process of performing the trial as a choice between proceeding to the next state in the schedule, say *j*, or remaining in the current state *i*: 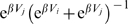. However, in the task with random cues this policy would give a 50% error rate, which is never observed. The motivation to perform at all, therefore, cannot be framed as a decision process of the choice type. In mathematical terms, *P*(*m*|*i*) can only depend on the current state and be an increasing function of its value. A softmax function, 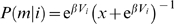, where the parameter *x*>0 may be required for proper normalization, is a natural choice [6],[29]. We have provided additional evidence for this choice because of its ability to explain detailed aspects of the behavior (Figure 4). The actual value of *x* is immaterial and could be tuned to maximize the agreement with the data in each dataset. However, we found one free parameter (*β*) to be sufficient, and we set *x* = 1 in Equation 1: this reduces the number of degrees of freedom and thus offers the most parsimonious account of the data. The same principle would demand *β* to be the same in both Equation 1 and in 
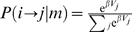
, although it need not be.

Regarding the general policy Equation 12, note that ∑*_j_ P_ij_*≤1, since *P*(*m*|*i*)≤1: the sum over *j* now represents the percentage of correct trials. Also, note that *j* can also be state *i*, if the agent is given the choice of remaining in the current state as a result of executing the corresponding action correctly. In such a case, the probability of remaining in state *i* is formally the product of the probability of leaving the state times the probability of choosing to return to it. This possibility is relevant if, for example, error trials need not be repeated. Thus, remaining in the current state can be the consequence of a choice (with probability *P*(*i*→*i*|*m*)·*P*(*m*|*i*)), or the consequence of an error (with probability *P*(*m̅*|*i*)), if error trials must be repeated. These two possibilities need to be kept conceptually distinct. If, after an error, the agent is positioned in a different state, the self-link in Figure 7A and B is replaced by a link to the corresponding state (with transition probability *P*(*m̅*|*i*)). In general, *P*(*i*→*i*|*m*) and *P*(*m*|*i*) depend on the same value *V_i_*, but in different ways. All values are learned with the same learning rule, which might be the standard TD method, or its generalization introduced here to account for the schedule length effect. If the latter method is used together with the full policy Equation 12, a schedule length effect for the error rates will emerge also in the choice-schedule tasks of Figure 6 (not shown).

Finally, this framework can be generalized to the case of transitions in continuous time. So far, transitions (including errors) could only happen at discrete time steps indexed by trial number, an adequate simplification for the purpose of this study. Formally, this means that *P_ij_* = *P_ij_*(*τ*), where *τ* is the trial number. However, one could define an arbitrary time unit *t*, much smaller than *τ*, so that each trial (or any event of duration longer than *t*) can unfold in time, like in the “serial compound representation” implementations of RL models (see, e.g., [30],[31]). Each transition would thus occur at a variable time, *P_ij_*(*t*) = *P*(*i*→*j*|*m*)·*P*(*m*|*i*;*t*), depending on *P*(*m*|*i*;*t*). In this scenario, at each time step *t* the agent “decides” to make a transition depending on *P*(*m*|*i*;*t*), and when it does, the final state of the transition is regulated by *P*(*i*→*j*|*m*), which may also depend on time, if the nature of the decision problem requires it. An error trial could be defined accordingly. For example, in the reward schedule, where reaction times are of the order of 300–600 ms, *t* could be chosen to be, e.g., 20 ms and the requirement of performing a correct trial would translate into a transition occurring between *t*_GO_+10*t* and *t*_GO_+50*t*, where *t*_GO_ marks the time onset of the GO signal. A lack of transition within this window is counted as an error: the agent is held in a “null” state for the duration of the inter-trial interval, during which no transitions are allowed, and then repositioned into the previous state at the beginning of the next trial. This implementation also allows the introduction of reaction time as the time elapsed since *t*_GO_ and when a transition occurs. It is predicted that the larger the motivational value, the faster the reaction time, and the smaller the number of errors, as observed in the experiments [17], [22]–[25].

## Discussion

In reward schedule tasks, monkeys make substantially more errors in validly cued unrewarded trials than in rewarded trials. The number of errors decreases with reward proximity. Also, the error rates are typically smaller in trials equally distant from reward, but belonging to longer schedules (schedule length effect; Figure 2). Both of these features disappear and monkeys make fewer errors in the absence of valid cues.

The monkeys do not maximize the amount of reward over the smallest number of trials, violating a principle requiring maximization of reward over time, and also violate the principle of invariance in trials equally far from reward, especially penultimate trials (Figure 2). This behavioral pattern occurs in most monkeys, thus it is robust and reliable. It only occurs after the meanings of the cues are learned, and persists over long periods (months or years despite constant practice in the task). Therefore, it should not be construed as maladaptive simply because it violates the principles of reward-maximization and invariance. Since the monkeys were allowed to work until they stopped by themselves, it can be inferred that they would get a sufficient amount of liquid reward, and were simply not interested in maximizing the amount over time.

We have argued that the monkeys' behavior is a direct consequence of learning the motivational values attached to each trial by using the cues. Either randomizing the cues or damaging the rhinal cortex prevents the formation of this typical error rates pattern [26],[32], and damaging orbitofrontal cortex blunts it [33]. In the model introduced here, the motivational values of the schedule states arise through trial-and-error learning and lead to suboptimal behavior. In its basic form, i.e., with *σ* = 0, this model can be described as TD-learning for solving the value prediction problem [6],[27]. The standard RL approach is usually concerned with the development of behavioral strategies that adapt towards optimality, and less often with the simpler value prediction problem, i.e., the problem of learning to predict the long term return obtainable starting from each behavioral state and following a given policy. Our interpretation of the RL method used in this work follows this thread, because the policy (the performance function Equation 1) is fixed and is not modified by the learning algorithm (of course its arguments, the values, are). This has been called “learning with an indirect actor” by Dayan and Abbott [34]. The particular policy used for the reward schedule departs from previous accounts because it depends on the value of the current state only. This is one of two core departures of our model from existing ones (e.g., [35],[36]). The second is the modification of the learning rule so as to capture the schedule length effect.

### Properties and Predictions of the Model in the Reward Schedule Task

In our model, a single algorithm explains the differential behavior with valid and random cues. Assuming that the average value of the schedule states is a measure of overall motivation, the model predicts that the overall motivation is similar in the valid and random cue conditions. The difference in performance in the two paradigms is a consequence of the non-linear (sigmoidal) shape of the performance function Equation 1 (cf. Figure 4C). The finding that the same overall level of motivation leads to different patterns of error rates with valid and random cues is not built into the model but is an emergent property of the learning process.

The context-sensitive model also predicts that, although the behavior appears to be the same in all terminal trials, terminal trials may acquire different values (see, e.g., Equation 9). This difference is not reflected in the behavior since the latter depends on both the values (which might be different) and the performance function (Equation 1), which tends to remove value differences in the high value region (Figure 4C). In this region the performance function (or its complement) is almost flat and slight differences in value will be unlikely to produce observable differences in error rate.

The context-sensitive behavior is also an emergent property of the model. The model does not change the definition of the schedule states to accommodate their contextual meaning. Valid cues come to “label” the schedule states via predictive learning. The basic model translates these labels into a pattern of motivational values and error rates which only depend on reward proximity, and thus are the same in penultimate trials. This symmetry is broken in the context-sensitive model as a consequence of generalizing the temporal difference so as to look backwards as well as forward, and not through a redefinition of the schedule states.

It might seem at first that the model does not take into account the cost of performing a trial, i.e., the cost of releasing the bar at the GO signal. In fact, this cost could be interpreted as the origin of the residual, non-zero error rate given by the performance function (Equation 1) when the values are maximal (approximately, the error rate in validly-cued rewarded trials). It is also possible to implement this cost so as to affect the values of each state, *V*(*S*)→*V*(*S*)−*c*, where *c* stands for cost. However, since the cost of the action is the same for all trials, it could not account for the differential error rates in different schedule states.

### Inadequacy and Generalization of Standard TD Learning

Our analysis unveils the inadequacy of standard TD learning for the reward schedule task. The general statement can be proved that it is not possible to capture the schedule length effect with RL methods inspired to TD learning, including TD(*λ*) [27], if these only take into account the values of trials remaining in the current schedule (cfr. Equation 4; see Materials and Methods for details). Thus, for a method based on temporal differences to capture the schedule length effect, its learning prescription must have access either to the value of a past trial in the current schedule, as proposed in this manuscript, or to the value of a trial belonging to a different schedule, a method that is not clear how to generalize beyond the reward schedule task.

The predictions of the context-sensitive model are the same as standard TD learning in a wide class of other tasks involving choice, where the values of states at decision nodes apply equally to whatever outcome of the decision. In simple choice tasks (cf. Figure. 5), both models predict a preference for more probable rewards, either always—under a greedy policy—or with occasional, temporary reversals of preference when the policy allows exploratory behavior—like the softmax function Equation 11. In the choice-schedule task of Figure 6A, the context-sensitive model predicts the same preference as the standard model. With schedules comprising more than two trials, choice preference of one model can be mapped into the choice preference of the other by readjusting the value of the discount rate *γ* appropriately. Thus the context-sensitive model, although heuristic in its derivation, appears to be a generalization of standard TD learning: it predicts the same behavior in tasks where human and animal subjects tprefer the choice leading to more probable or larger rewards; but it also predicts the violation of the principle of invariance occurring in the reward schedule task, not captured by the standard model; and it predicts the “procrastination-like” behavior of monkeys in the same task. The latter is to be generally expected in tasks requiring a step-wise approach to reward, where the willingness to act in each single trial exerts a powerful influence on the behavior. More work is required to characterize fully the mathematical properties of the model, and explore its possible derivation from well-defined principles as is customary in the fields of Machine Learning and RL, which is beyond the scope of this work.

### Extension to General Markov Decision Processes

The reward schedule and choice tasks represent two particular cases of general MDPs where the problem of making a decision can be factorized into two sub-problems, the motivation to perform at all, and the selection of one among alternative choices given the motivation to act. We have used the strategy of dividing this general problem into two parts: we have analyzed the behavior as driven by motivational value using the reward schedule task, and the behavior as driven by choice preference using choice tasks. In both cases, we have compared the standard and the novel TD model using the same policy for both. These two components are simply multiplied in general MDPs, where by definition both the motivation to act and choice selection can occur.

Our results indicate that only in the choice selection problem does the actor-critic architecture of RL [6],[37] potentially have a significant role. In the actor-critic architecture, the RL problem is solved by two related “structures,” one responsible for performing the action (the actor), the other responsible for criticizing those actions based on evaluative feedback (the critic). Actor-critic architectures usually lead to policies that maximize the long-term return, and thus they seem to have only a small role in the reward schedule task. If an underlying actor-critic is present, its effectiveness in producing optimal control might be blunted by an opposing force deriving from the purely motivational nature of the problem encountered in this task, i.e., whether or not to comply with its demands. Indeed, we have shown that it is sufficient for the critic to assess the value of the current trial and use it to direct the level of engagement in the task, without the need for a more specialized actor structure as would be required for action selection [38]. Instead, the process of valuation of several alternatives, potentially leading to different courses of actions and rewards as it typically occurs in general decision problems, could benefit more from an actor-critic organization of the behavior.

### Related Work

The extension of RL to capture the fundamental role of motivation in reinforcement schedules is currently a major challenge for the field, and other authors have also considered how to include motivation in RL [14],[15]. These authors focused on incorporating overall drive (e.g., such as degree of hunger or thirst) so as to describe how habitual responses can be modified by the current motivational level, which is, in turn, assumed to influence generalized drive through sensitivity to average reward levels [39]. In the reward schedule, however, we focused on how motivation orients behavior in a trial-specific, not generalized, manner. In such a case, an alternative solution to ascribing errors to a decreased level of motivation is Pavlovian-instrumental competition, which has been used to explain suboptimal behavior [16]. Applied to the reward schedule task, this solution would posit that error trials would result from the competition between the negative valence of the valid cue associated to an unrewarded trial (acquired through a Pavlovian-like mechanism), and the incentive to perform the same trial correctly to reach the end of the schedule and obtain reward. This interpretation is supported somewhat by the fact that the visual cues have no instrumental role in the reward schedule (they are neither triggers nor instructors of correct behavioral actions). The schedule length effect, however, escapes explanations in terms of Pavlovian-instrumental competition and would still have to be taken into account. Instead, the single motivational mechanism put forward in this work accounts for all the aspects of the behavior; has a natural interpretation in terms of learned motivation to act, however originated; and can be extended to general MDPs.

A dependence on the value of the preceding state implemented in our learning rule suggests an explanation of the schedule length effect as a history effect. When environmental cues are not perfect predictors of the availability of resources, monkeys' decisions about where to forage depend on past information like the history of preceding reinforcements [40], or stored information about recent trends in weather [41]. Lau and Glimcher [28] have found that past choices, in addition to past reinforcements, must be taken into account to predict the trial-by-trial behavior of rhesus monkeys engaged in a choice task resulting in matching behavior. However, contrary to the statistical description of Lau and Glimcher [28], past information in our model bears an effect on the learning rule, not directly on the action selection process, and it does so through the value of the previous state, as opposed to past reinforcements or past choice history. Taken together, these findings point to some form of sensitivity to preceding actions and visited states (or their values) in primates' foraging behavior, and the schedule length effect might be a side effect of such a mechanism, perhaps also present in other forms of reinforcement learning.

### Relation to Neural Data

Current theories of reinforcement learning posit that dopaminergic neurons code for a prediction error signal analogous to *δ* in our model and in TD learning in general [30]. Data from dopaminergic neurons of monkeys performing a reward schedule task, however, are not in sufficient accord with the predictions of such theories [24]. For example, one prediction is that *δ*, and therefore dopamine neurons, after sufficient training should cease to respond to predicted reward, and this was not observed. Recent developments [42],[43] rule out that this could be the consequence of the small temporal jitter around reward delivery. Despite the incongruence with the assumed role of dopamine neurons as signaling some form of prediction error, there is clear evidence of the involvement of dopamine in learning. In the reward schedule, the importance of dopamine D2 receptors for learning the meaning of new valid cues has been demonstrated in perirhinal cortex [26], and Ravel and Richmond [24] have argued that salient events may drive dopaminergic neurons, whose activity may be required for enhancing the connection of the stimulus with its prediction in perirhinal cortex.

The contextual impact of the organization of the task in schedules has been found in the event-related responses of neurons in all neural structures investigated thus far in the reward schedule task, except perhaps for neurons of the area TE [22]. The brain area where the neural modulation with schedule state is most apparent is the anterior cingulate cortex [44]. One third of the neurons recorded in this area keep track of the progress through the schedule in the Valid Cue condition, and could reflect the (motivational) value of the schedule states and their being linked to one another in a chain of states culminating in the rewarded trial. Another candidate structure for the representation of the schedule states is the perirhinal cortex, whose neurons become selective for the meaning of the visual cues, as opposed to, e.g., TE neurons' responses that are locked to their physical identity [22].

In some brain regions, neuronal responses are different in trials of different schedules that might be regarded as homologous, particularly last trials of different schedules. Dopamine neurons [24], perirhinal neurons [22] and ventral striatum neurons [23] respond differently to valid cues in last trials (predicting the same reward, but in different schedules). This is reminiscent of the phenomenon that the context-sensitive model assigns different values to terminal trials belonging to different schedules.

Neurons of the basolateral complex of the amygdala often have differential post-cue activity in first trials [25]. In these neurons another, different effect related to the organization in schedules has also been observed: these neurons increase their activity in the pre-cue period before the beginning of each schedule. No pre-cue activity was observed in the Random Cue condition, supporting the hypothesis that pre-cue activity is related to the contextual imprint of the task's organization in schedules [25]. This activity could be related to a context-sensitive representation of the values of the states, either in the amygdala itself, or in areas connected to the amygdala like perirhinal cortex [22], anterior cingulate cortex [44] and ventral striatum [23], where the schedule state meaning of valid cues is strongly represented. The possibility of a more specific role of the amygdala for the emergence of the schedule length effect will be considered later when discussing the analogous phenomenon of “framing” in humans.

Finally, there is evidence for the role of the primate striatum in learned action selection, with some authors [45] proposing for its ventral part coding for the values of states (reminiscent of the critic in actor-critic RL methods), and its dorsal part coding for the values of actions and for action selection (reminiscent of the actor [11],[13],[45]; but see [38]). In the reward schedule, the largest population of ventral striatum neurons which are responsive around the time of bar release, do so in rewarded trials, with the second larger population being responsive in all trials [23]. Comparison of latency and periods of peak activity between these neurons and neurons of the orbitofrontal cortex suggest that the latter are better positioned for representing the reward contingency and thus for guiding action, whereas the former are more related to executing the action [46]. This role is usually ascribed to more dorsal regions of the striatum, but the involvement of the ventral striatum is conceivable in the reward schedule, given the simple action selection required (it amounts to the timely execution of the bar release in all contingencies), and it is compatible with our model, where the probability of a correct bar release is based solely on the value of the current state and not on action values.

### Interpretation of the Schedule Length Effect: Framing and Sunk Cost

In the context-sensitive model, the mechanism responsible for the schedule length effect leads to the violation of invariance. The violation of this principle was invoked by Tversky and Kahneman in their description of “framing” [18],[19]. Framing describes the process whereby the choice made is influenced by the manner or context in which the choice is presented. Thaler [47] and Tversky and Kahneman [18] showed that humans often act as if they kept separate accounts for gains and losses, rather than estimate the total value. A consequence of keeping separate accounts is that the manner in which a problem is cast, in terms of gains, of losses, or of total value influences choices. For example, people purchasing two items, costing respectively $15 and $125, are more willing to put an effort (for example by driving to another store) to save $5 when this is presented as a discount on the $15 item, than when presented as a discount on the $125 item, even though the total saving is the same [18],[47],[48]. Similarly, monkeys are willing to put more effort in a trial if the total effort to get there had been larger, even though this does not affect the upcoming reward. A “minimal account” would consider only the proximity to reward, whereas the behavior of the monkeys shows that a combination of minimal (reward proximity) and topical (workload) accounts affects their motivation when facing a reward schedule. From this point of view, reward proximity could be seen as a property defining the state (in accord with Equation 7), much like the $5 discount defines the saving in the example above, independently of the item to which it is nominally attached. In both cases, it is the comparison with some truly contextual attribute that assigns a different motivational value to the same action. Thus, especially on penultimate trials, the length of the schedule seems to exert a contextual effect on the monkeys' motivation analogous to framing. A more direct, preliminary example of framing in monkeys has been reported recently [49] using a task similar to one previously used with starlings [50].

The schedule length effect is also reminiscent of the so-called “sunk cost” effect [20],[21],[51],[52], “a maladaptive behavior that is manifested in a greater tendency to continue an endeavor once an investment in money, effort or time has been made” [21]. The sunk cost phenomenon comes in different varieties and with different interpretations (to the point of having different names, like “Concorde effect,” “cognitive dissonance,” “work ethics,” see [20] for a review), some of which come close to framing. In one interpretation, sunk cost derives from the violation of the principle that “a prior investment should not influence one's consideration of current options; only the incremental costs and benefits of the current options should influence one's decision” [20]. The similarity with the schedule length effect and with the previous discussion about its interpretation in terms of framing seems obvious. A relevant example is Experiment 2 of Arkes and Blumer [21]. In this experiment, three groups of patrons were sold season tickets for the Ohio University Theater at three different prices, and those who purchased tickets at either of the discounted prices attended fewer plays during the season. In this case, the money spent at the beginning of the season influenced the patrons' choice to attend the plays.

It could be argued that, in the reward schedule task, the cost of performing trials is not strictly a “sunk” (wasted) cost, as it would be if the monkeys had to start the schedule anew after each error trial. However, this would only be a minor difference with other instantiations of sunk cost effects; and it could similarly be argued that the money spent in Experiment 2 of Arkes and Blumer [21] is not a wasted cost, since it is necessary to attend the plays.

Various explanations of sunk cost and framing have been proposed. Arkes and Ayton [20] explain the sunk cost fallacy as an overgeneralization of the “don't waste” rule, since based on their review of the literature, the effect is not unambiguously present in lower animals, and is not found in children [20]. Even if the schedule length effect can legitimately be interpreted in terms of sunk cost or framing, we think that this is unlikely to be the correct explanation. A better explanation may be linked to emotional factors. A functional imaging study [53] points to an important emotional component in the susceptibility to frames in humans. This study found the susceptibility to framing linked to amygdala activations, with the ability to resist the frame linked to activation of the orbital and medial frontal cortex. Similarly, we believe that there is a strong emotional component responsible for the monkey's reaction to unrewarded cues (leading to larger error rates), and possibly for the schedule length effect. Thus, a connection between this emotional component and parameter *σ*, which quantifies the schedule length effect in our model, could be speculated on the basis that a larger *σ* implies a larger schedule length effect, in the same way as a larger emotional component would imply a stronger susceptibility to framing [53]. We do not reject this idea as a possibility, but our data are not sufficient evidence for it.

Our model does make a clear prediction in one case where framing has been found, i.e., in the increase in preference due to training with a larger cost [51], a case of state-dependent learned valuation. In this experiment, starlings preferred to choose stimuli which had previously associated with a larger effort (16 1-m flights vs. four 1-m flights) to obtain an otherwise identical reward. Since this paradigm pitted two reinforcement schedules of different length against each other, there are obvious similarities with our reward schedule task. Indeed, it would be possible to run a similar test in monkeys by associating different cues to terminal trials in different schedules (e.g., cue H for the longer schedule and cue L for the shorter), and then test the monkeys' preference in a choice task where there is no cost (or equal cost) to obtain the same reward from two sources, one cued with H, the other with L. Would the monkey prefer the cue associated during training with the longer schedule, as found in starlings [51]? Our model predicts exactly this. Because of the accumulation of previous values, the values of terminal trials are larger in longer schedules in the model with *σ*>0. Assuming that in the choice task preference depends on the same learned values, the source of reward cued by H (previously associated with the longer schedule) would be preferred. This also means that our model implies state-dependent learned valuation when the state of the animal is defined by the cumulative effort expended to obtain the reward.

We stress, however, that our learning model is not meant to be a general model of the effects that frames, or sunk costs, have on humans and animals. For example, Pompilio et al [52] offer additional evidence of state-dependent valuation in an invertebrate (the grasshopper), but in their case the state of the animals at the time of learning is defined by their nutritional state (e.g., more or less hungry) as opposed to their expended cost. They found that the grasshoppers, in a later choice task with equal cost, prefer the food experienced when in a lower nutritional state during learning. We do not see a connection between this finding and the schedule length effect, or the role of the parameter *σ*. This should not be surprising. As Pompilio et al. [52] point out, there may be more than a single mechanism responsible for state-dependent valuation, depending on the animal and, in the same animal, depending on the paradigm used for training.

### Conclusions

In the heuristic modification of TD learning introduced in this work, the schedule length effect emerges spontaneously from the sensitivity to the immediately preceding trial, leading to the violation of the invariance principle. Since this principle is violated in instances of framing and sunk cost effects, we have interpreted the monkeys' behavior using the framing and sunk cost analogies, even though monkeys might not be susceptible to framing or sunk cost the way humans are. We are not aware of alternative RL models predicting the violation of the principle of invariance.

## Materials and Methods

### Subjects and Behavioral Paradigm

In this work we collate the behavioral data from earlier studies on monkeys (*Macaca mulatta*) tested in the reward schedule task [22]–[26],[32],[44]. In all of these studies, randomly interleaved schedules of one, two or three trials must be completed to obtain a reward. In *n* = 3 monkeys, schedules with 4 trials were also used. A trial begins when the monkey touches a bar (Figure 1A), causing the appearance of a visual cue. Four hundred milliseconds later a red dot (WAIT signal) appears in the center of the cue. After a random interval of 500–1500 ms the dot turns green (GO signal). The monkey is required to release the touch-bar between 200 and 800 ms after the green dot appeared, in which case the dot turns blue (OK signal), and a drop of liquid reward is delivered 250 to 350 ms later. If the monkey releases the bar outside the 200–800 ms interval after the GO signal, an error is registered, and no reward is delivered. To start, monkeys are trained on this simple color discrimination task, with or without the presence of a cue, and are rewarded for every correct trial. When performance reaches criterion (at least 75% correct), reward schedules start. Each reward schedule is a sequence of 1, or 2, or 3, …, or *N_s_* trials, where *N_s_* is the maximal schedule length for that session (3 or 4; see Figure 1B for a 2-trial schedule). All schedules are selected with equal probability, and within a schedule error trials must be repeated until performed correctly. Only correct terminal trials are rewarded. After a correct terminal trial, a new schedule is selected pseudo-randomly. Each schedule state is labeled by the pair {*τ*,*s*}, where *τ* = 1, 2, …, *s* stands for trial and *s* = 1, 2, …, *N_s_* stands for schedule. Terminal trials have *τ* = *s*. Trials of different schedules representing the same schedule fraction (e.g., 1/2 and 2/4) are considered different schedule states, even though they might have been associated to the same visual cue (Valid Cue condition, see below). Different cue sets have been used in different studies [17], [22]–[26],[32],[44],[54], producing similar behavioral results. For the data shown in Figure 2, collected by Sugase-Miyamoto and Richmond [25] (panel *A*) and Shidara and Richmond [44] (panel *B*), horizontal bars with different brightness were used as cues, and the cues were brighter as the schedule progressed. Other cue sets have also been used. Some, still based on cue brightness, had the opposite relationship between brightness and proximity to reward, e.g., cues were darker towards the end of the schedule, as, e.g., in Figure 1 [22],[24],[32],[54], to ensure that the behavior of the monkeys was not biased by the direction of brightness. Other cue sets were based on bar length [26],[32]; still others consisted of unique stimuli like, e.g., Walsh patterns [26], to establish that the behavior was not a consequence of having a sensory attribute (like length or brightness) increasing or decreasing with proximity to reward. The typical behavioral patterns that are the main focus of this work were similar across individual experiments and cue sets.

In the paradigm with random cues, the same visual stimuli are present, but each stimulus is selected pseudo-randomly with equal probability in each trial (Random Cue condition). In such a case, there is no relationship between cues and schedule states, although the schedules are still in effect.

The monkeys were not taught the “rules” of the reward schedule task but were simply exposed to it. The behavior reported in Figure 2 emerges spontaneously, typically within a week of the first exposure, depending on the monkey (in some cases, it emerges on the very first day), and it generalizes rapidly (in less than 3 days) to different cue sets.

### Data Analysis and Statistics

For each monkey, the error rates were calculated as the ratio of the total number of incorrect trials (in all sessions) to the total number of trials for each schedule state. Differences in error rates across schedule states were tested with a *?* ^2^ test of the contingency table obtained from the numbers of correct and incorrect trials (confidence was taken at the 5% level). Pair-wise comparisons of the error rates in different schedule states were tested with the Marascuilo procedure after a significant *?* ^2^ test [55]. If the *?* ^2^ test is significant at the *α* level, the Marascuilo procedure [55],[56] provides a confidence interval of 100(1−*α*)% for each pair-wise difference of error rates |*p_i_*−*p_j_*|, where *p_i_* = *e_i_*/*n_i_* is the error rate in schedule state *i*, and *e_i_*, *n_i_* are, respectively, the number of error trials and total trials in schedule state *i*. The Marasquilo confidence interval on |*p_i_*−*p_j_*| is given by 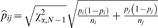. In this formula, 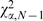 is the critical value of *?* ^2^ with *N*−1 degrees of freedom at *α* level of significance (the point of the distribution which leaves an area of α in the upper tail of the distribution). *N* is the number of different schedule states. Schedule states with |*p_i_*−*p_j_*|>*p*ˆ*_ij_* are significantly different at the *α* level.

A sign test [57] was run on the number (*n*_+_) of monkeys showing better performance in penultimate trials belonging to longer schedules, as compared to the number (*n*_−_) of monkeys where either the inverted pattern, or no difference, was observed. The “exact” binomial probability for *n*_+_ successes in *n*_+_+*n*_−_ trials was used.

Reaction times were defined as the time elapsed since the appearance of the GO signal and the bar release, and, as reported previously, were generally shorter in trials more proximal to reward [17], [22]–[25]. Reaction times had a similar relationship to schedule states as did error rates. Since they provide no new qualitative interpretation, they were not analyzed further.

### Model Fitting

For each monkey, the theoretical error rates (*p*_th_) were fitted to the experimental error rates (*p*_ex_) by minimizing a weighted sum of squares, 
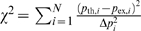
, where the sum goes over all schedule states in both the Valid and Random Cue conditions, and 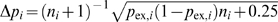 [58]. The reason for this choice is that the interval 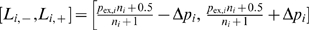 is approximately a 68% confidence interval around *p*_ex,*i*_ based on Wilson's “score” equation [59],[60], and (*L_i_*_,+_−*L_i_*_,−_)/2 = Δ*p_i_*. The theoretical error rates were given by Equations 2, 9, and 10. The minimization of *?* ^2^ was accomplished with a full factorial search of the best-fit values for parameters *β*, *γ*, and *σ* of Equations 2, 9, and 10.

### Solution of the Model

The formula Equation 6 of the main text for the equilibrium values of the basic model is exact only in the absence of errors, otherwise the values are smaller and are given by the self-consistent, recursion formula:
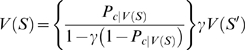
(13)Here, *S*′ is the next state in the schedule, *P_c_*_|*V*(*S*)_≡P(*c*|*V*(*S*)) is the probability of correct performance in (current) state *S*, conditioned on the value of that state, *V*(*S*). *V*(*S*) appears also on the left hand side, and for this reason the formula defines *V*(*S*) only implicitly. If *S* is a terminal trial, *γV*(*S*′) must be replaced by *r* in Equation 13. By iteration, Equation 13 gives
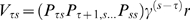
(14)where 
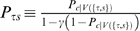
 to simplify the notation. This set of equations must be solved self-consistently for *V_τs_* as the *P_τs_* depend on *V_τs_*. Under the optimal policy of not making any errors, i.e., with each *P_c_*_|*V*_≡1 independently of *V* instead of Equation 1, Equation 14 becomes the explicit solution given by Equation 6 reported in the main text.

Equation 13 can be derived as follows: at equilibrium, *V*(*S*) is the average of the value obtained after an error (*V_e_*, occurring with probability *P_e_*_|*V*_≡1−*P_c_*_|*V*_) and the value obtained after a correct trial (*V_c_*, probability *P_c_*_|*V*_), conditioned on current average value being *V*, i.e.
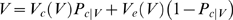
(15)with *V_c_*(*V*) = *V*+*α*(*γV*′−*V*) and *V_e_* = *V*+*α*(*γV*−*V*). The last two equations are simply the update equation for *V* after a correct and an incorrect trial respectively; *V*′≡*V*(*S*′) is the value of the next schedule state after a correct trial (*γV*′ must be replaced by *r* in terminal trials). Solving Equation 15 for *V* gives Equation 13.

The same procedure, though more involved algebraically, gives the values in the context-sensitive model:
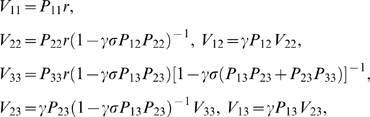
where *P_rs_* is defined as for the basic model. This system of equations must be solved self-consistently for the values *V_rs_*. In the absence of errors, each *P_rs_* = 1 and Equations 9 of the main text follow. We have checked with simulations that the approximate solution given by Equations 9 gives a good approximation to the correct values on our dataset of monkeys' data. For this reason, Equations 2 and 9 were used to estimate the theoretical values when fitting the theoretical error rates to the experimental error rates.

In the Random Cue condition, the cues define the states of the model. The model learns the values of the cues using the same algorithm specified by Equations 1, 3, and 8, with *S_t_*≡*cue_t_*. The next cue is selected at random with equal probability for all cues if the trial is performed correctly, otherwise the current cue remains as the next. We set *δ* = *r_t_*+*σV*(*cue_t_*_−1_)−*V*(*cue_t_*) in terminal trials, in keeping with the rule adopted with valid cues. The average value of random cues can be obtained by averaging the update equation over all trial types that produce a different temporal difference *δ*, obtaining
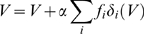
(16)i.e., ∑*_i_ f_i_δ_i_*(*V*) = 0, where *V* is the sought average value, *f_i_* is the average frequency with which trial *i* occurs, and *δ_i_* is the temporal difference in trial *i*. In the basic model, it is sufficient to distinguish three trial types: correct terminal trials, incorrect terminal trials, and non terminal trials. The frequency (*f*) of correct terminal trials is *N*_+_ *P_c_*_|*V*_, where *N*_+_ is the average fraction of rewarded trials, equal to the number of schedules divided by the number of schedule states. In correct terminal trials the temporal difference is *δ* = *r*−*V*. Incorrect terminal trials occur with frequency *N*_+_(1−*P_c_*_|*V*_) and have *δ* = −*V*; non-terminal trials occur with frequency 1−*N*_+_ and generate a temporal difference *δ* = (*γ*−1)*V*, whether the trial is correct or not. Replacing these values in Equation 16 and solving for *V* gives
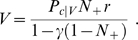
(17)


Equation 17 defines *V* only implicitly and must be solved self-consistently to give the exact value of *V*. For the small error rates usually encountered with random cues, Equation 17 is well approximated by its version in the absence of errors (*P_c_*_|*V*_ = 1 for any *V*), i.e. 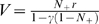. Note how *V* increases with *γ* and is constrained between the average collected reward *N*_+_ *r* (for *γ* = 0) and *r* (for *γ* = 1). Setting γ = 0 (value at which *V* is minimal) is the same as assuming that the next cue is always unknown and its value is zero (cfr. Equation 4). This implies that having some expectation about the next state, even a random expectation as for the random cues, increases the values and hence the motivation to perform correctly.

The context-sensitive model can be solved in a similar way, with in addition non-first trials to be taken into account. The final result is 
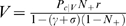
, from which Equation 10 of the main text follows under the approximation of small error rates, i.e., *P_c_*_|*V*_≈1. Similar results are obtained in the case of post-reward expectation, where the value of the next state after a rewarded trial is not set to zero, as shown in a later subsection.

Since it is required that *V*>0, this result requires (*γ*+*σ*)(1−*N*_+_)<1, or (*γ*+*σ*)<(*N_s_*+1)(*N_s_*−1)^−1^. This inequality is never violated in the basic model (where *σ* = 0), but it might be, and must be imposed, in the context-sensitive model, especially for long maximal schedule lengths. Similar restrictions coming from the values of valid cues also apply (e.g., *σ*<1/2*γ* from Equation 9).

### Insufficiency of Forward-Type Methods of Temporal Difference Learning (Including TD(*λ*))

Here we show that it is not possible to obtain values dependent on schedule length (like in the context-sensitive model) by using a standard TD learning rule, which considers only future trials within the current schedule. The most general such rule can be written as 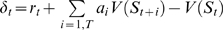, where the coefficients {*a_i_*}*_i_*_ = 1,2,…,*T*_ may depend on pre-reward number (i.e., the number of trials remaining before reward), but not on schedule length. *t*+*T* is the time at which the terminal trial is reached: when *S_t_* is a terminal trial, the states *S_t_*_+*i*_ are not defined and their values are set to zero. It is more convenient to express the values as a function of the number, *n*, of trials remaining before reward (“0” being the terminal trial), conditioned on schedule length being *s*, *V*(*n*|*s*), as in Equation 7 of the main text. At equilibrium (*δ_t_* = 0) one has *V*(1|*s*) = *a*_1_ *V*(0|*s*). Since *V*(0|*s*) = *r* does not depend on *s*, *V*(1|*s*) does not depend on *s*, which in turn implies that *V*(2|*s*) = *a*_1_ *V*(1|*s*)+*a*_2_ *V*(0|*s*) does not depend on *s*, and so on. It follows by induction that *V*(*n*|*s*) does not depend on *s* for all pre-reward numbers *n*, and for any value of the coefficients *a_n_* (some of which may vanish). This result holds also in the case of post-reward expectation, where the value of the next state after a rewarded trial is not set to zero and the forward terms in the series ∑ *a_i_V*(*S_t_*_+*i*_) are taken to the (*T*+1)th term. As shown later, all values are re-scaled by a constant factor which does not depend on *s*, leaving the above argument unchanged.

It follows from this argument that, to obtain the schedule-length effect, it is necessary either to look backwards at the values of previous trials in the same schedule (as in the context-sensitive model of the main text), or to take into account trials belonging to different schedules [61]. The notable TD(*λ*) rule (see, e.g., [6]), that has been suggested to be implemented by dopamine neurons of rats [62], considers only forward trials within the current schedule, and therefore cannot produce the contextual effect due to schedule length. In fact, here we show that for the reward schedule, the equilibrium values in the TD(*λ*) rule are the same as those obtained with the basic model. In TD(*λ*), all forward trials within a schedule are considered, weighted by imminence. Formally, when in state *S_t_* at time *t*, the TD term is evaluated as
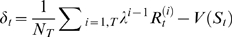
(18)where the sum is over all states remaining until the terminal one (reached after *T* steps). *λ* is a parameter between zero and one; *N_T_*≡1+*λ*+λ^2^+…+*λ^T^* ^−1^ is a normalization factor; and 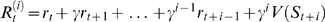 is the *i*-steps-ahead prediction starting from *S_t_*. (If *λ* = 0, Equation 18 reduces to *δ_t_* = *r_t_*+*γV*(*S_t_*_+1_)−*V*(*S_t_*), the basic model of the main text.) The values are updated in the usual way: *V_t_*_+1_ = *αδ_t_*. In the reward schedule it is 
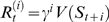
 (only the terminal trial is rewarded), and Equation 18 reads
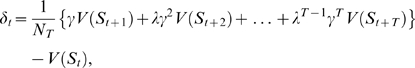
(19)where *t*+*T* is the time at which the terminal trial is reached. The solution to *δ_t_* = 0, with *δ_t_* given by Equation 19, is the same as for the basic rule (Equations 3 and 4 of the main text), i.e., *V*(*S_t_*_+*i*_) = *γ^T^* ^−*i*^ *r*, or *V*(*τ*,*s*) = *γ^s^* ^−*τ*^ *r* if *S_t_*≡{*τ*,*s*}, as can be proved, e.g., by direct substitution. This was confirmed in simulations of TD(*λ*)-learning of the reward schedule implemented through the use of eligibility traces, an alternative approach to TD(*λ*) (see [6] for details).

### Solution of the Model with Post-Reward Expectation

So far, the value of the next state at the end of each schedule had been to set to zero. In other words, the learning rule following a rewarded trial is *δ_t_* = *r_t_*+*σV*(*S_t_*_−1_)−*V*(*S_t_*), which written in this form applies to all cases, including the case of random cues and the basic model (where *σ* = 0). As said in the main text, this is the common choice in RL [6]. Here we show that behavior predicted by the model does not change if we assign a positive value to the next state (“post-reward expectation”). The reason is that, in a terminal trial, the next trial is not known and thus the same value must be assumed independently of current schedule. The actual value is immaterial, but for the sake of argument we shall make a choice. In the Random Cue condition, the current value of any cue chosen at random will do; in the Valid Cue condition, since the only available information is that the next state will be one of the initial trials {1,*s*}, the average value of all first trials will be taken, i.e., 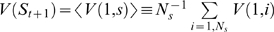. It can be shown that the average value of each state is increased by a constant factor (1−*γϑ*)^−1^, where *ϑ* is the ratio of the value of the state post-reward to the value of rewarded trials. For the value chosen above (average of first trials), 
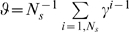
 (note that this choice gives *γϑ*<1). Similarly, the value of random cues changes from 
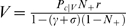
 to 
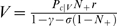
. Thus, there is no qualitative difference with respect to the case of no post-reward expectation of the main text. A similar argument also shows that the qualitative behavior does not change if *σ*>0 in first trials of each schedule in the context-sensitive model.

## References

[1] Bell DE, Raiffa H, Tversky A (1988). Decision making: Descriptive, normative, and prescriptive interactions.

[2] Schweighofer N, Shishida K, Han CE, Okamoto Y, Tanaka SC (2006). Humans can adopt optimal discounting strategy under real-time constraints.. PLoS Comput Biol.

[3] Kirby KN (1997). Bidding on the future: evidence against normative discounting of delayed rewards.. J Exp Psychol Gen.

[4] Dickson JW (1978). The effect of normative models on individual and group choice.. Eur J Soc Psychol.

[5] Niv Y, Joel D, Dayan P (2006). A normative perspective on motivation.. Trends Cogn Sci.

[6] Sutton RS, Barto AG (1998). Reinforcement learning: An introduction.

[7] Simon HA (1986). Rationality in psychology and economics.. J Bus.

[8] Schoemaker PJ (1982). The expected utility model: its variants, purposes, evidence and limitations.. J Econ Lit.

[9] Arrow KJ (1982). Risk perception in psychology and economics.. Econ Inq.

[10] Tversky A, Kahneman D (1986). Rational choice and the framing of decisions.. J Bus.

[11] Samejima K, Ueda Y, Doya K, Kimura M (2005). Representation of action-specific reward values in the striatum.. Science.

[12] Pessiglione M, Seymour B, Flandin G, Dolan RJ, Frith CD (2006). Dopamine-dependent prediction errors underpin reward-seeking behaviour in humans.. Nature.

[13] Haruno M, Kawato M (2006). Different neural correlates of reward expectation and reward expectation error in the putamen and caudate nucleus during stimulus-action-reward association learning.. J Neurophysiol.

[14] Dayan P, Balleine BW (2002). Reward, motivation, and reinforcement learning.. Neuron.

[15] Dayan P, Dietterich TG, Becker S, Ghahramani Z (2002). Motivated reinforcement learning.. Advances in Neural Information Processing Systems 14..

[16] Dayan P, Niv Y, Seymour B, Daw ND (2006). The misbehavior of value and the discipline of the will.. Neural Netw.

[17] Bowman EM, Aigner TG, Richmond BJ (1996). Neural signals in the monkey ventral striatum related to motivation for juice and cocaine rewards.. J Neurophysiol.

[18] Tversky A, Kahneman D (1981). The framing of decisions and the psychology of choice.. Science.

[19] Kahneman D, Tversky A (1984). Choices, values, and frames.. Am Psychol.

[20] Arkes HR, Ayton P (1999). The sunk cost and Concorde effect: are humans less rational than lower animals?. Psychol Bull.

[21] Arkes HR, Blumer C (1985). The psychology of sunk cost.. Organ Behav Hum Decis Process.

[22] Liu Z, Richmond BJ (2000). Response differences in monkey TE and perirhinal cortex: stimulus association related to reward schedules.. J Neurophysiol.

[23] Shidara M, Aigner TG, Richmond BJ (1998). Neuronal signals in the monkey ventral striatum related to progress through a predictable series of trials.. J Neurosci.

[24] Ravel S, Richmond BJ (2006). Dopamine neuronal responses in monkeys performing visually cued reward schedules.. Eur J Neurosci.

[25] Sugase-Miyamoto Y, Richmond BJ (2005). Neuronal signals in the monkey basolateral amygdala during reward schedules.. J Neurosci.

[26] Liu Z, Richmond BJ, Murray EA, Saunders RC, Steenrod S (2004). DNA targeting of rhinal cortex D2 receptor protein reversibly blocks learning of cues that predict reward.. Proc Natl Acad Sci U S A.

[27] Sutton RS (1988). Learning to predict by the methods of temporal differences.. Mach Learn.

[28] Lau B, Glimcher PW (2005). Dynamic response-by-response models of matching behavior in rhesus monkeys.. J Exp Anal Behav.

[29] Daw ND, O'Doherty JP, Dayan P, Seymour B, Dolan RJ (2006). Cortical substrates for exploratory decisions in humans.. Nature.

[30] Schultz W, Dayan P, Montague PR (1997). A neural substrate of prediction and reward.. Science.

[31] Montague PR, Dayan P, Sejnowski TJ (1996). A framework for mesencephalic dopamine systems based on predictive Hebbian learning.. J Neurosci.

[32] Liu Z, Murray EA, Richmond BJ (2000). Learning motivational significance of visual cues for reward schedules requires rhinal cortex.. Nat Neurosci.

[33] Simmons JM, Minamimoto T, Murray EA, Richmond BJ (2007). Lesions of orbitofrontal cortex in rhesus monkeys disrupt assessments of outcome value as a function of cost. Neuroscience Meeting Planner.

[34] Dayan P, Abbott LF (2005). Theoretical neuroscience: computational and mathematical modeling of neural systems.

[35] Egelman DM, Person C, Montague PR (1998). A computational role for dopamine delivery in human decision-making.. J Cogn Neurosci.

[36] McClure SM, Daw ND, Montague PR (2003). A computational substrate for incentive salience.. Trends Neurosci.

[37] Joel D, Niv Y, Ruppin E (2002). Actor-critic models of the basal ganglia: new anatomical and computational perspectives.. Neural Netw.

[38] Atallah HE, Lopez-Paniagua D, Rudy JW, O'Reilly RC (2007). Separate neural substrates for skill learning and performance in the ventral and dorsal striatum.. Nat Neurosci.

[39] Niv Y, Daw ND, Dayan P (2005). How fast to work: Response vigor, motivation and tonic dopamine.. Neural Information Processing Systems.

[40] Sugrue LP, Corrado GS, Newsome WT (2004). Matching behavior and the representation of value in the parietal cortex.. Science.

[41] Janmaat KR, Byrne RW, Zuberbuhler K (2006). Primates take weather into account when searching for fruits.. Curr Biol.

[42] Fiorillo CD, Schultz W, Newsome WT (2007). The temporal precision of reward prediction in dopamine neurons. Neuroscience Meeting Planner.

[43] Kobayashi S, Schultz W (2007). Temporal discounting in the activity of dopamine neurons during a Pavlovian task. Neuroscience Meeting Planner.

[44] Shidara M, Richmond BJ (2002). Anterior cingulate: single neuronal signals related to degree of reward expectancy.. Science.

[45] O'Doherty J, Dayan P, Schultz J, Deichmann R, Friston K (2004). Dissociable roles of ventral and dorsal striatum in instrumental conditioning.. Science.

[46] Simmons JM, Ravel S, Shidara M, Richmond BJ (2007). A comparison of reward-contingent neuronal activity in monkey orbitofrontal cortex and ventral striatum: guiding actions toward rewards.. Ann N Y Acad Sci.

[47] Thaler R (1980). Toward a positive theory of consumer choice.. J Econ Behav Organ.

[48] Savage LJ (1954). The Foundations of Statistics.

[49] So N, Stuphorn V (2007). Framing effects on decision-making under risk in macaque monkeys. Abstract Viewer/Itinerary Planner.

[50] Marsh B, Kacelnik A (2002). Framing effects and risky decisions in starlings.. Proc Natl Acad Sci U S A.

[51] Kacelnik A, Marsh B (2002). Cost can increase preference in starlings.. Anim Behav.

[52] Pompilio L, Kacelnik A, Behmer ST (2006). State-dependent learned valuation drives choice in an invertebrate.. Science.

[53] De Martino B, Kumaran D, Seymour B, Dolan RJ (2006). Frames, biases, and rational decision-making in the human brain.. Science.

[54] Simmons JM, Richmond BJ (2008). Dynamic changes in representations of preceding and upcoming reward in monkey orbitofrontal cortex.. Cereb Cortex.

[55] NIST/SEMATECH (2006). e-Handbook of Statistical Methods: Online Publication.. http://www.itl.nist.gov/div898/handbook/prc/section4/prc474.htm.

[56] Marascuilo LA (1971). Statistical Methods for Behavioral Science Research.

[57] Zar JH (1999). Biostatistical Analysis.

[58] La Camera G, Rauch A, Thurbon D, Luscher HR, Senn W (2006). Multiple time scales of temporal response in pyramidal and fast spiking cortical neurons.. J Neurophysiol.

[59] Brown LD, Tony Cai T, DasGupta A (2001). Interval estimation for a binomial proportion.. Stati Sci.

[60] Meyer PL (1965). Introductory Probability and Statistical Applications.

[61] La Camera G, Liu Z, Pritchett DL, Richmond BJ (2005). Modeling the behavior of monkeys in reward schedules with context-dependent and adaptive reinforcement learning. Abstract Viewer/Itinerary Planner.

[62] Pan WX, Schmidt R, Wickens JR, Hyland BI (2005). Dopamine cells respond to predicted events during classical conditioning: evidence for eligibility traces in the reward-learning network.. J Neurosci.

